# The M1/M2 Test System for Determining Macrophage Phenotypes

**DOI:** 10.3390/ijms27167488

**Published:** 2026-08-21

**Authors:** Daria Surkova, Polina Vishnyakova, Viktoriia Kiseleva, Andrey Elchaninov, Timur Fatkhudinov

**Affiliations:** Research Institute of Molecular and Cellular Medicine, Peoples’ Friendship University of Russia (RUDN University), 6 Miklukho-Maklaya Street, Moscow 117198, Russia; surkova-de@rudn.ru (D.S.);

**Keywords:** macrophages, test system, M1, M2, phenotype

## Abstract

Macrophages are highly plastic innate immune cells that integrate diverse microenvironmental cues to adopt pro-inflammatory (M1) or anti-inflammatory (M2) functional states, which critically influence the pathogenesis of infectious, autoimmune, inflammatory, and malignant diseases. This review provides an overview of current concepts of macrophage ontogeny, functional heterogeneity, and disease-associated phenotypes, with a specific focus on experimental approaches used as M1/M2 test systems for macrophage phenotyping. The scope of the review encompasses commonly used experimental models, induction protocols for M1- and M2-like polarization, and key readouts, including gene-expression signatures and metabolic parameters. Particular emphasis is placed on reporter-based platforms (luciferase, BRET, fluorescent nanoparticle probes), label-free biophysical methods such as electrical impedance monitoring and metabolic profiling, and their application to dynamic, real-time assessment of macrophage phenotype in the context of tumor microenvironments and chemotherapeutic exposure. The potential of integrating reporter systems with single-cell omics, spatial transcriptomics, and patient-derived ex vivo platforms is considered, with a view to transforming M1/M2 test systems into clinically oriented assays capable of tracking macrophage programs during therapy and supporting the development of macrophage-targeted diagnostics and treatments.

## 1. Introduction

Macrophages are innate immune cells that detect typical pathogen molecules and initiate immune responses (phagocytosis, cytokine secretion etc.). These cells are distributed throughout tissues for rapid mobilization and response to pathogens and injury. The totality of macrophages and monocytes is traditionally united into the so-called mononuclear phagocyte system. A relatively modern view of this system was formulated in the 1970s by Ralph van Furth [1]. The core of the proposed concept was that monocytes are continuously produced in the red bone marrow and migrate via the bloodstream into organs, where they differentiate into macrophages and dendritic cells, which compensate for the loss of the corresponding populations due to cell death [1]. However, as new data accumulated, it became clear that such populations of tissue macrophages as microglia, Kupffer cells of the liver, Langerhans cells of the epidermis, and some others, under normal conditions, practically do not depend on a bone marrow origin [2]. All these macrophage populations are self-maintaining and are formed in the prenatal period from progenitor cells that are not related to hematopoietic cells of the red bone marrow [3,4,5].

They were named tissue-resident macrophages (TRMs). In addition, currently dendritic cells are not classified as belonging to the monocyte-macrophage lineage [6,7].

The role of TRMs in tumor progression is remarkable [8]. Under the influence of the tumor microenvironment, this type of cells acquires a so-called tumor-associated phenotype, and their ability to proliferate contributes to an increase in the pool of tumor macrophages [9]. At the same time, proliferating TRMs of embryonic origin exhibited a fibrotic transcriptional profile indicating their role in the production and remodeling of molecules in the extracellular matrix [10]. Thus, in the context of tumor progression, TRMs contribute to tumor progression and create immunosuppressive niches, rather than directly destroying the tumor [11].

Macrophages possess a wide repertoire of functions that can be rather conventionally divided into immune (protective) and non-immune ones [5]. As cells of innate immunity, macrophages are necessary for the elimination of foreign microorganisms due to their own phagocytic activity, as well as through the recruitment of monocytes and granulocytes into the infectious focus by synthesizing chemoattractants [2]. In addition to nonspecific defense, macrophages are capable of initiating adaptive immune responses owing to their ability to present antigens [12]. There is no consensus in the scientific literature on this issue. Many researchers believe that the only cells involved in antigen presentation are dendritic cells, and that macrophages, despite expressing MHC-II, are not capable of antigen presentation [6,7,13].

The list of so-called non-immune functions of macrophages is quite extensive and depends on the specific tissue-resident population: participation in synaptogenesis in nervous tissue [14], facilitation of electrical conduction in cardiac muscle tissue [15], regulation of proliferation in the epidermis [16], involvement in maintaining surfactant homeostasis [17], regulation of hematopoiesis [18], and others. In addition, the involvement of macrophages in the development of pathological processes has been described. Particular importance is attributed to macrophages in tumor development [19]. Clinical interest is also attracted by the participation of macrophages in reparative processes [20,21]. Besides local activity, a distant influence of macrophages on cutaneous wound healing has been described. Thus, activation of peritoneal macrophages is associated with stimulation of skin regeneration. This is presumably due to the secretion of fibronectin into the bloodstream by peritoneal macrophages [22]. The results of such studies support the view of macrophages as a system-forming factor underlying inter-organ interactions [23].

Thus, if one ignores the specific functions of a given tissue-resident population, macrophages exhibit a certain dualism: they can both promote inflammation and resolve it. Based on this, by analogy with the Th1/Th2 immune response, the so-called M1/M2 paradigm emerged, describing the functional characteristics of macrophages and their involvement in inflammation [24].

According to the M1/M2 dichotomy, macrophages are divided into M1 (pro-inflammatory polarization type) and M2 (anti-inflammatory polarization type) [24,25]. The pro-inflammatory properties of macrophages are activated upon recognition of pathogen-associated molecular patterns (PAMPs), followed by the production of pro-inflammatory cytokines, including *TNF-α*, IL-1β, IL-6, and the recruitment of larger numbers of leukocytes [25]. Polarization of macrophages toward an anti-inflammatory phenotype occurs under the influence of cytokines IL-4 and IL-13, as well as IL-10 and IL-33. Macrophages with an anti-inflammatory phenotype are characterized by high production of IL-10 and TGF-β, involvement in the synthesis and remodeling of extracellular matrix components, and stimulation of angiogenesis [26].

As data accumulated, subtypes began to be distinguished within each functional macrophage type. The M2 macrophage group turned out to be particularly heterogeneous and was subdivided into M2a, M2b, and M2c depending on activating factors, levels of CD163 and CD206 expression, and IL-10 secretion [27,28]. Subsequently, however, a concept emerged of a continuous in vivo phenotypic continuum in which the extreme states are M1 or M2 [29]. Evidence for this has been obtained; for instance, in the study by Specht et al., it was shown that a population of mature macrophages displays a continuum of phenotypes even in the absence of inducers [30]. Based on this, many researchers have concluded that the terminology of the M1/M2 dichotomy should be abandoned altogether [2,31]. Despite such radical views emphasizing the oversimplification of the model, the terms themselves have become entrenched in the scientific literature and are frequently used [32]. The existence of a phenotypic continuum considerably complicates the work of researchers, who are forced to define the M1/M2 phenotype based on the predominance of the corresponding marker at the time of the study. The simplification imposed by the M1/M2 dichotomy of macrophages represents, on the one hand, a substantial limitation of all approaches and test systems that attempt to unambiguously define macrophage phenotype, and on the other hand, a challenge that remains to be addressed in the context of macrophage research. The development of multicolor and multiparametric assays as M1/M2 test systems for determining the direction of polarization is likely to become the next step in this field. The examples cited in the review could well be combined to address this task.

### Significance of Studying Macrophage Phenotypes

By now, a substantial number of studies have demonstrated the role of macrophages in the development of various pathological conditions. Consequently, macrophages represent an attractive target for the development of new diagnostic and therapeutic approaches for diverse diseases [33]. Furthermore, macrophages themselves can serve as one of the options for cell therapy [34]. Further progress in this field is impossible without a deep understanding of the mechanisms underlying the formation of macrophage functional phenotypes and the methods for their assessment [35].

As already noted, the phenotype of macrophages involved in the development of a particular disease represents a continuum. Nonetheless, one can distinguish groups of pathologies (conventionally) in which an M1 pro-inflammatory state predominates and, accordingly, an M2 anti-inflammatory state of macrophages.

A classic example of nosologies with a pronounced M1 macrophage phenotype, especially in the acute phase, is infectious diseases. This is most clearly demonstrated in the lungs. In infection with influenza A virus, a large number of monocyte-derived macrophages accumulate in the lungs, exhibiting high sensitivity to PAMPs due to elevated expression of multiple TLRs [36]. These macrophages produce high levels of IL-6 [37,38]. A similar phenomenon is observed in inflammatory bowel diseases. The intestinal wall accumulates large numbers of CD14^hi^ monocyte-derived macrophages with a pro-inflammatory phenotype [39]. Interestingly, inflammation persisting in the intestinal wall leads to changes in bone marrow hematopoiesis. Monocytes that already possess a pro-inflammatory phenotype are released from the red bone marrow into the bloodstream. It is assumed that this occurs due to IFNγ produced by the intestinal wall [40].

Autoimmune diseases are a group of nosologies in which macrophages play a prominent role in pathogenesis [41]. In such cases, macrophages usually exhibit an M1 phenotype. For example, in patients with systemic lupus erythematosus, higher expression of CD40L (CD40 ligand) has been observed, which increases even further under the influence of LPS [42]. Treating monocytes from healthy donors with plasma obtained from patients with systemic lupus erythematosus leads to a marked increase in the expression of M1 markers CD80, CD83, and CD86 [43]. One of the markers of the M1 state of macrophages is synthesis of the chemokine CCL5 [29]. In patients with rheumatoid arthritis, an association has been found between disease development and the level of CCL5+ macrophages in the synovial membrane [44]. In systemic sclerosis, a clear-cut association between either an M1 or M2 macrophage state and disease development has not been identified. Macrophages display a broad spectrum of markers associated with both pro- and anti-inflammatory phenotypes [45]. Similar data have been obtained in multiple sclerosis [46,47].

Macrophages in tumors are the numerically dominant cells of the myeloid lineage [48]. Most tumor-associated macrophages differentiate from infiltrating monocytes. Tissue-resident macrophages may also be found in tumors, and their number depends on tumor-specific features [49]. Macrophages participate in all stages of tumor progression. Malignant tumors are a classic example of nosologies in which the macrophage state is skewed toward the M2 direction [19,33]. Nevertheless, tumor-associated macrophages display substantial heterogeneity that does not fully conform to the M1/M2 paradigm [48]. Moreover, if one examines the macrophage phenotype in the most common tumors, only some of their markers are related to the M1 or M2 state [50]. For example, in breast cancer and hepatocellular carcinoma, expression of the M2 markers CD163 and CD206 is characteristic [51]. These proteins participate in the infiltration of tumors by CD8+ lymphocytes, which is a manifestation of macrophage-dependent antitumor protection [19]. At the same time, some studies have demonstrated the prognostic significance of these markers in malignant disease. High levels of soluble CD163 and CD206 in the plasma of patients with acute myeloid leukemia are associated with poor prognosis [52]. Similar data have been obtained for CD206 in solid tumors [53]. Hence, monitoring the dynamics of macrophage immunophenotypes may be of great importance.

The aim of this review is to critically summarize current knowledge on macrophage polarization and to systematize the experimental approaches that constitute an M1/M2 test system for determining macrophage phenotypes, including standardized induction protocols, phenotypic markers, and readout techniques, with an emphasis on their applications, limitations, and translational potential in cancer, autoimmune and infectious diseases.

## 2. Literature Search Strategy

Relevant literature was identified through systematic searches of the Google Scholar and PubMed databases. Searches were performed using combinations of the following keywords: “macrophage polarization”, “macrophage THP-1 polarization”, “M1 macrophages”, “M2 macrophages”, “tumor-associated macrophages”, “reporter system”, “fluorescent reporter”, “bioluminescence”, “real-time imaging”, “engineered macrophages” and “macrophage phenotype”.

Only articles published in English and containing original experimental data or comprehensive reviews were included. Additional relevant publications were identified by examining the reference lists of selected articles.

## 3. The M1/M2 Test System

### 3.1. Description of the M1/M2 Test System

The balance between M1 and M2 phenotypes in the macrophage population plays a crucial role in the pathogenesis and progression of numerous diseases. The predominance of M2-like protumoral macrophages contributes to immune suppression, tumor growth, and resistance to chemotherapy, making macrophage polarization an important target for both basic research of mechanisms of tumor progression and drug testing for selection of an individual treatment path.

In recent years, one of the major challenges in biomedicine has been the investigation of macrophages in the context of tumor progression, particularly for tumor-associated macrophages (TAMs) that are located in the tumor microenvironment. Numerous studies have demonstrated that several chemotherapeutic agents used in standard anticancer treatment protocols may induce unwanted effects by shifting the M1/M2 macrophage balance toward the protumoral M2 phenotype.

The majority of studies of macrophage phenotypes are focused on determining the distribution of macrophage subsets within a particular population at a specific time point. However, macrophages are characterized as highly plastic cells and their phenotype can dynamically change over time in response to microenvironmental signals. Therefore, investigation of macrophage polarization in the context of anticancer chemotherapy requires monitoring of macrophage phenotypic changes in real time. This is important due to the dynamic nature of the tumor microenvironment and its influence on macrophage physiology and functional state.

Another important aim of biomedicine nowadays is the rapid assessment of macrophage phenotype in response to various chemotherapeutic agents. Such test systems may facilitate high-throughput screening of chemotherapeutic compounds for creating a platform for selecting a personalized treatment path aimed to minimize undesirable effects of treatment.

Recent publications from the last 5–7 years describe the development of reporter systems and other analytical platforms for monitoring macrophage phenotypes in response to different stimuli highlighting the interest in this field. These findings prove the relevance of developing novel macrophage phenotype detection systems in the era of personalized medicine.

### 3.2. Methodology for Isolating and Culturing of Macrophages

Methods of isolation and culturing of macrophages for studies depend on the tissue of interest, intended use in further research etc. Due to the presence of multiple cell sources, several methodologies have been developed. There are some most common macrophage isolation techniques such as isolation of bone marrow–derived progenitor cells, isolation of tissue-specific macrophages and isolation of peripheral blood monocytes.

Bone-marrow derived macrophages (BMDMs) are widely used as a primary macrophages model in murine studies. BMDMs are typically isolated from the femurs and tibias of mice by flushing of the bones with sterile medium [54]. After isolation cell suspension is cultured in the presence of recombinant macrophage colony-stimulating factor (M-CSF). An alternative way is adding an L929-conditioned medium as a source of M-CSF [54]. Under these conditions hematopoietic progenitors differentiate into macrophages within 6–8 days [55].

Macrophages derived from peripheral blood monocytes are a widely used model for studies involving human cells. Peripheral blood mononuclear cells (PBMCs) are isolated by density gradient centrifugation using Ficoll or similar Lympholyte, then monocytes can be isolated by magnetic sorting using CD14-specific antibodies [56]. PBMCs-derived monocytes differentiate into macrophages by culturing with M-CSF for 6–8 days. After differentiation, macrophages may be used for further experiments.

Primary macrophages derived from peripheral blood monocytes are one of the most relevant in vitro models for macrophage studying because they keep a lot of functional characteristics of macrophages in vivo and are easy to obtain for a large number of experiments and research [29]. When working with monocytes, a M-CSF is used to differentiate progenitor cells into mature macrophages [57]. The resulting macrophages can subsequently be exposed to various activation factors to study further cell differentiation and changes in their physiology in tissues. One of the most substantial advantages of macrophages obtained from monocytes using M-CSF is the homogeneity of the macrophage population that responds to polarization stimuli [58].

The most challenging protocols of macrophages obtaining are for isolation of tissue-specific macrophages. Peritoneal macrophages are one of the easiest tissue macrophages to obtain and are commonly isolated by peritoneal lavage with sterile buffer [59]. Protocols are different for different types of tissue macrophages, and they are usually used for murine studies.

Macrophages’ behaviour in culture depends on their microenvironment during culturing. Successful culturing requires attention to nutrients, growth factors, polarization signals, and physical conditions stability to maintain viability. After isolation macrophages are cultured in a medium containing all necessary nutrients for cells such as glucose, amino acids, inorganic salts, serum-derived growth factors for proper cell growth, proliferation, and differentiation. Sometimes antibiotics and antimycotics are added to minimize the risk of contamination and false results. It is also important to maintain stable temperature, pH value, humidity, and CO_2_ concentration conditions.

In the context of developing a test-system for tracking macrophage phenotype in real time, especially for humans, the most suitable source of macrophages are macrophages derived from peripheral blood mononuclear cells. Peripheral blood collection is a minimally invasive procedure and allows repeated sampling from individual donors for long studies and development of personalized treatment trajectory. Another advantage is the possibility enriching monocytes by cell sorting, for example using CD14-based magnetic selection, to generate relatively pure and homogeneous M-CSF-derived macrophage populations [60]. Such a macrophage population may be used for quantitative assessment of macrophage phenotype dynamics under the influence of chemotherapeutic agents due to their steady responses to polarization stimuli.

## 4. Key Markers for Identifying Macrophage Phenotypes

Macrophage polarization is accompanied by changes in the expression of cell surface receptors, transcription factors, secretion of cytokines and chemokines (Table 1). In vitro polarization by different agents also causes those changes. Accurate characterization of macrophage phenotype cannot be achieved using a single marker and requires the analysis of multiple complementary markers illustrating various aspects of macrophage biology. Therefore macrophage phenotype is usually characterized using a combination of surface markers, secreted cytokines and chemokines and gene expression signatures. The choice of analysing markers depends on cell origin, experimental model and accessible method of analysis.

### 4.1. Surface Markers

Surface markers are frequently assessed by flow cytometry, immunocytochemistry, immunohistochemistry and ELISA. M1 macrophages are characterized by high expression levels of CD80, CD86, CD40, CD64, CCR7, TLR2, TLR4, MHC-II, HLA-DR [61,62,63,64]. In contrast, alternatively activated M2 macrophages are characterized by high expression levels of CD163, CD206, CD204, CD209 [62,63]. Among these markers, CD80 and CD86 are the most common for pro-inflammatory M1 macrophages and CD163 and CD206 for anti-inflammatory M2 macrophages [65].

CD80 and CD86 are membrane proteins expressed by M1 macrophage cells and are used for identification of pro-inflammatory macrophage. They are stimulatory molecules on antigen presenting cells and play a major role in activation of T cells, their proliferation and production of cytokines [66]. Activation of T cells include interaction with CD28 and CTLA-4 [66]. Increasing CD80 and CD86 expression demonstrates a pro-inflammatory profile in macrophages. Increasing of CD80/86 expression is also associated with IFN-γ induced macrophage polarization [67].

CD163 is a haptoglobin-hemoglobin scavenger receptor that is involved in the resolution of inflammation through activation of anti-inflammatory signaling pathways by scavenging pro-oxidative and pro-inflammatory hemoglobin with further induction of heme oxygenase-1 and the generation of anti-inflammatory metabolites that leads to the resolution of inflammation [68]. It also binds the tumor necrosis factor-*TNF-α* (*TNF-α*), viruses and pathogenic bacteria. The expression of CD163 is upregulated in response to interleukin-10 (IL-10) [69].

CD206 is the mannose receptor that is also involved in antigen presentation and endogenous molecule clearance [70]. Mannose receptor is also involved in mediation signaling pathways and induce tissue remodeling and angiogenesis and therefore is one of the most widely used markers of macrophage M2 polarization [71]. CD206 expression level increases in response to IL-4 and IL-13.

### 4.2. Secreted Factors

Alternative variant to determine macrophage phenotype is using cytokines and chemokines that are secreted by macrophages to regulate other immune cells’ activity as markers of polarization. The difference in cytokines and chemokines profile in M1 and M2 macrophages can be explained by metabolic differences between these two functional states. M1 macrophages secrete pro-inflammatory cytokines such as *TNF-α*, IL-6, IL-12, IL-1α, IL-1β, IL-18, IL-23 and IFN-γ while M2 macrophages secrete anti-inflammatory cytokines such as TGF-β, IL-10, IL-4, IL-13, IL-33 [62,72,73].

For macrophage phenotype determination chemokines are also used. There are several subfamilies of chemokines: CXC, CC and CX3C with different structural features [74]. In CC-chemokines two cysteines are arranged sequentially. CXC-chemokines have 1 amino acid residue that separates two cysteines. CXC type chemokines contain three amino acids between the first two cysteines. For pro-inflammatory M1 macrophages CXCL1, CXCL3, CXCL5, CXCL8, CXCL9, CXCL10, CXCL11, CXCL16; CCL2, CCL3, CCL5, CCL8, CCL15, CCL19 and CX3CL1 chemokines are used [73,75]. For anti-inflammatory M2 macrophages CCL1, CCL2, CCL13, CCL14, CCL17, CCL18, CCL22, CCL24 and CCL26 are used [62,75].

Of all these soluble mediators *TNF-α*, IL-1β, IL-6, and IL-12 are most commonly used as indicators of M1 polarization, whereas IL-10, TGF-β and CCL18 are widely used as markers of M2 macrophages.

*TNF-α* is a strong pro-inflammatory cytokine that plays one of the most important roles in the inflammation process, pathogen elimination and has an antitumor effect [76]. IL-6 may promote tumor cell survival and migration, which leads to tumor development and progression and macrophage polarization toward M2-like phenotype [77]. Also, IL-6 induces the expression of the key angiogenic factors HIF-1α and VEGF. For IL-1β was described a similar effect: enhancement of angiogenesis and tumor growth [77].

IL-10 is one of the strongest anti-inflammatory cytokines produced by M2 macrophages that plays a significant role in inflammation resolution. IL-10 inhibits transcription regulators related to M1 polarization and this positive feedback loop helps to maintain anti-inflammatory M2 phenotype [72]. TGF-β plays an important role in deposition and remodeling of extracellular matrix after injuries and also takes part in immune suppression [78]. CCL18 is also known as a chemokine produced by M2 macrophages and for CCL18 produced by tumor-associated macrophages inducing of chemoresistance was previously shown [79].

### 4.3. Other Soluble Factors

In addition to the secreted factors that define M1 and M2 macrophages, several other soluble factors contribute to macrophage functions by regulating angiogenesis, extracellular matrix remodeling, and tissue repair. These molecules are often not considered as canonical polarization markers but provide important information about the functional state of macrophages and the surrounding microenvironment.

An important class of macrophage-derived soluble factors consists of matrix-remodeling enzymes such as metalloproteinases (MMPs). Secretion of MMP-1, -3, -7, -10, -25 is increased in pro-inflammatory M1-macrophages, whereas secretion of MMP-8, -9, -11, -12, FIZZ1, Ym1/2 is increased in alternatively activated M2-macrophages [63,80].

Macrophages additionally secrete local lipid mediators that regulate both the initiation and resolution of inflammation. For M1-macrophages production of leukotriene B4 (LTB4), and thromboxane B2 (TXB2) was higher than for M2-macrophages. Production of maresin 1 (MaR1), resolvins D5 and E2 (RvD5, RvE2), protectin D1 (PD1), lipoxins A4 (LXA4) was increased in anti-inflammatory macrophages compared to pro-inflammatory macrophages.

## 5. Induction Protocols for M1 and M2 Polarization

Plasticity, that is, the ability to adapt the functional phenotype in response to signals from the surrounding microenvironment, is a unique property of macrophages that allows them to perform diverse and even opposing functions, such as stimulating inflammation, resolving it, or promoting tissue repair [81]. The process by which macrophages adopt different functional states is called polarization [82]. This delicate process involves a chain of molecular transformations, from the activation of receptors and transcription factors to metabolic reprogramming and epigenetic stabilization of the polarized phenotype [83,84,85].

While in vivo polarization is a continuum rather than a binary state, in vitro induction protocols using defined cytokine and pathogen-associated stimuli remain the standard tool for generating reproducible M1- and M2-like populations for mechanistic study [86]. A persistent challenge in ex vivo macrophage polarisation research is that the different research groups have historically used inconsistent stimuli, cell sources and marker panels to define them. An analysis of several studies on in vitro macrophage polarization revealed both consensus and significant disagreement regarding the choice of cell models, stimulation protocols, and interpretation of results. The focus was on the THP-1 cell line as the most widely used model, as well as comparisons with primary macrophages and the U937 cell line.

First and foremost, all the studies reviewed agree that the THP-1 cell line exhibits a pronounced predisposition to the M1 phenotype. This is confirmed by both direct functional assays and transcriptomic profiling [87,88]. THP-1 macrophages actively respond to classic M1 stimuli (IFN-γ + LPS), demonstrating high expression of proinflammatory genes and markers such as CD40 and CD64, which Immunobiology identifies as the most specific for human M1 cells. At the same time, the ability of THP-1 to polarize toward M2 is assessed extremely controversially. The most radical position is taken by the Maria Rynikova study, which, based on whole-genome RNA sequencing, concluded that none of the common protocols (with IL-4, IL-13, or vitamin D3) can reliably generate M2 macrophages from THP-1. This directly contradicts an earlier paper in the E.W. Baxter studies, which proposed standardized protocols for obtaining M(IL-4) and M(IL-10) phenotypes in THP-1 cells, although the authors acknowledge that these cells do not fully recapitulate the transcriptional signature of primary macrophages. Thus, the major contradiction is that different assessment methods (real-time PCR vs. RNA sequencing) yield different conclusions about the success of M2 polarization, while a more sensitive transcriptomic approach reveals an incomplete M2 program in THP-1 cells.

An important aspect that is often overlooked is the impact of the maturation (differentiation) stage of monocytes into macrophages. Daphne Y.S. Vogel et al. demonstrated that the use of M-CSF or GM-CSF for the maturation of primary monocytes predetermines their subsequent response to polarizing stimuli: M-CSF creates a moderate anti-inflammatory background (M2 bias), while GM-CSF, conversely, induces a pro-inflammatory bias (M1 bias) [61]. This fact is directly relevant to studies on THP-1, where differentiation is typically achieved with phorbol 12-myristate 13-acetate (PMA). Liu et al. optimized this particular step, finding that a concentration of 80 ng/mL PMA is optimal for THP-1 differentiation based on CD14 marker expression; however, their work does not address subsequent polarization [89]. However, the very fact that PMA protocols are standard makes THP-1 even more “programmed” for an M1 response, which is consistent with the data of Nascimento et al., who showed that THP-1 outperforms the U937 cell line in reactive oxygen species production and phagocytic activity—properties characteristic of M1.

Regarding marker availability, there is a high degree of agreement. Daphne Y.S. Vogel et al. clearly identifies CD40 and CD64 as the most reliable surface markers of M1, while CD163 and the mannose receptor (CD206) are key markers of M2 in humans [61]. These data echo transcriptional profiles from the E.W. Baxter studies, where the M(IL-4) phenotype is characterized by the expression of CCL17, CCL26, and CD200R, while the M(IL-10) phenotype is characterized by CD163, C1QA, and SEPP1 [90]. However, Maria Rynikova study questions even these established markers for THP-1, showing that their induction upon IL-4 stimulation is insufficient to classify cells as having a full M2 phenotype.

Thus, the presented studies demonstrate that the simple binary division between M1/M2, often used by researchers, is an oversimplification. This is despite the fact that, as early as 2014, Murray et al. proposed abandoning this rigid dichotomy and describing macrophages using three principles: cell source, stimuli used, and a set of markers [29]. This recommendation remains relevant today, as current data demonstrate a continuous spectrum of activation states, including hybrid and transitional phenotypes.

Based on the presented studies, it can be concluded that THP-1 is a reliable and convenient model for studying macrophage behavior in inflammatory responses, especially in combination with PMA + IFN-γ + LPS. However, to understand the role of macrophages in regenerative processes, it is preferable to use primary macrophages or U937 cells.

When choosing a differentiation protocol, it should be considered that M-CSF and GM-CSF provide different initial backgrounds, a factor that may influence the interpretation of polarization results.

## 6. Applications of the M1/M2 Test System

### 6.1. Understanding Immune Responses in Various Diseases

Macrophages play an important role in immunity and are involved in progression of various diseases. The balance between pro-inflammatory M1 macrophages and anti-inflammatory M2 macrophages often determines the outcome of inflammatory reactions and tissue repair processes.

A test system for phenotype determination can be helpful not only for testing the effectiveness of chemotherapeutic drugs but also for investigating the macrophage polarization under different pathological conditions in vitro. Commonly used methods such as flow cytometry, quantitative PCR and ELISA can show the distribution of phenotypes in a specific population at a specific moment. Due to high plasticity of macrophages it is important to monitor macrophage phenotype transitions in living cells in real time.

In infectious diseases, test systems may facilitate studies of pathogen-host interactions and identification of microbial factors influence on macrophage phenotype changes. TLR4 recognizes lipopolysaccharide (LPS) from Gram-negative bacteria, cryptococcal capsular components, and mycobacterial products, that launch signaling cascades that lead to the production of pro-inflammatory cytokines such as *TNF-α*, IL-1β, IL-6, and IL-12 [91]. TLR2 is the pattern recognition receptor that may recognise the largest variety of microbial ligands, including fungal peptidoglycans, the mycobacterial glycolipid lipoarabinomannan, and bacterial lipoproteins [92]. TLR9 in turn recognizes unmethylated CpG dinucleotides presented in bacterial DNA [93].

Bacterial infections may also be initiated by extracellular vesicles produced by bacterial cells after interaction that modulate macrophage phenotype, which in turn changes the phenotype of other macrophages nearby [94]. Real-time monitoring of macrophage activation states can therefore provide important insights into disease pathogenesis and mechanisms of evading the immune response.

In autoimmune and chronic inflammatory diseases, including rheumatoid arthritis, inflammatory bowel disease, multiple sclerosis, atherosclerosis, irregularity of macrophage plasticity contributes to persistent inflammation and tissue damage [95]. Also, macrophages play a significant role in tissue remodeling and regeneration so monitoring their phenotypic dynamics may be useful in regenerative medicine and tissue engineering [96].

### 6.2. Investigating Tumor Microenvironments

Tumor affects its microenvironment that leads to monocyte infiltration from peripheral blood, differentiation to macrophages and phenotype switch from pro-inflammatory M1 state to pro-tumoral M2 state that induce cancer cell proliferation, epithelial-to-mesenchymal transition, matrix remodeling for metastasis, neoangiogenesis, lymphangiogenesis that leads to rapid tumor growth and progression and worsening of the patient’s status [97].

Several widely used chemotherapeutic drugs, including docetaxel (Taxotere), cisplatin, doxorubicin (Adriamycin), and gemcitabine, have been reported to induce a protumoral M2 phenotype in TAMs, thereby contributing to the development of tumor cell chemoresistance [98]. The principal mechanisms underlying TAMs-mediated M2 polarization and chemoresistance include modulation of intracellular signaling pathways in macrophages, recruitment of additional immunosuppressive TAMs populations followed by their further protumoral polarization, activation of Th17-mediated immune responses that promote tumor development, and induction of anti-apoptotic programs in tumor cells. These changes contribute to reduction of sensitivity of tumor cells to chemotherapeutic agents and as a result tumor progression, therapeutic failure, and cancer recurrence.

### 6.3. Clinical Implications: Biomarkers for Disease Prognosis

Monocyte and macrophage functional state has become an important prognostic factor for a variety of pathological states and consequently macrophage phenotype dynamic change especially during inflammatory and tumor diseases often correlates with disease severity and clinical outcome.

In oncology, close attention is directed to the tumor-associated macrophages, which often acquire an M2-like phenotype usually characterized by expression of CD163 and CD206. In this regard macrophages are used as a prognostic marker by performing an immunohistochemical (IHC) analysis. For example, during IHC for tissue cores of representative tumor areas predominance of CD163 macrophages compared to CD68 macrophages was shown [99]. There are similar studies related to other types of cancer.

IHC analysis for CD163 and CD206 tumor-associated macrophages was performed for breast cancer patients. It was shown that CD163 TAMs were associated with high-grade tumors whereas CD206 TAMs were associated with increasing frequency of lymph node metastasis [100]. High stromal CD206 expression was more frequent than high intratumoral CD206 expression and for CD163 macrophages it was also shown that patients with high intratumoral CD163 expression and low stromal CD163 expression had the shortest relapse-free survival [100]. Similar data about macrophage influence on the cancer progression and poor prognosis was published for colorectal cancer, glioblastoma, oral squamous cell carcinoma [101,102,103].

For chronic inflammatory diseases such as rheumatoid arthritis, multiple sclerosis, autoimmune hepatitis and inflammatory bowel disease presence of MHC-II, IL-4 and *TNF-α* markers in peripheral blood is determined for prediction of the course of the disease [47].

## 7. Techniques Used in Existing Test Systems

The majority of modern studies investigating functional changes in macrophages a static approach to macrophage phenotype assessment dominates. Within this research structure, researchers evaluate the expression levels of specific markers associated with either M1 or M2 polarization at a single time point, thereby collecting only static data of the macrophage population state. One of the major limitations of endpoint analyses is the inability to characterize intermediate activation states through which macrophages pass during polarization processes. Macrophages are highly plastic cells and real-time monitoring of phenotypic changes is essential for understanding the consecutive events leading to M1 or M2 polarization. Dynamic monitoring is particularly important in studies of macrophage polarization induced by pharmacological agents, including chemotherapeutic drugs.

Bioluminescent reporter systems are widely used tools for monitoring macrophage phenotypes. The principle that is the basis of these approaches is bioluminescence: a luciferase gene is placed under the control of a promoter of the gene that is associated with a particular macrophage phenotype, resulting in luciferase expression and light emission following oxidation of luciferin in case of the promoter activation. In this context, luminescence intensity is proportional to promoter activity and therefore reflects expression levels of the gene of interest.

One of the major challenges associated with reporter protein-based systems, including reporters, is the relatively low transfection efficiency of primary macrophages. As a result, most studies have relied on monocytic or macrophage cell lines rather than primary cells. Although immortalized cell lines provide an overview into macrophage behavior under certain experimental conditions, findings obtained in vitro may not fully reproduce the behavior of native tissue-resident macrophages in vivo.

For example, mechanisms regulating expression of the M2 marker arginase-1 have been investigated in murine RAW264.7 macrophages using a reporter construct in which the luciferase gene was inserted downstream of the *Arg1* promoter. Following IL-4 stimulation, strong luminescence signals were detected in reporter cells, demonstrating the effectiveness of this reporter system for monitoring M2 polarization [104].

The use of an *Arg1*-luciferase bioluminescent reporter system has also been proposed as a method for detecting small tumors in vivo [105]. It is known that tumor-associated macrophages acquire M2-like phenotypes characterized by expression of arginase-1. Transplantation of genetically engineered macrophages carrying a luciferase gene under the control of the *Arg1* promoter into animals with tumors leads to macrophage migration to tumor focus and generation of detectable bioluminescent signals [105]. Using this assay, tumors with volumes as small as 25–50 mm^3^ could be detected. A similar strategy has been applied to detect different regulatory elements within the promoter of inducible nitric oxide synthase (*NOS2*), a classical M1 macrophage marker, using the murine macrophage cell line RAW264.7 [106].

One of the major limitations of bioluminescence-based reporter systems is the difficulty of tracking individual cells located deep in tissues. To overcome this challenge, Tedeschi and colleagues proposed the use of bioluminescence resonance energy transfer (BRET) combined with spectral phasor analysis for monitoring macrophage polarization [107]. In this study, two BRET reporters were cloned under the control of the promoters of *NOS2* (an M1-associated marker) and *STAT6* (an M2-associated marker). The *STAT6* promoter region was amplified from the p4xSTAT6-Luc2P plasmid, whereas the *NOS2* promoter was amplified from the pGL2-NOS2Promoter-Luciferase plasmid. Upgraded nanofluorophores CeNL (blue emission) and YeNL (yellow emission) were selected as BRET probes. This reporter system was validated in 3D collagen matrix models using RAW264.7 macrophages. M1 polarization was induced with LPS, whereas M2 polarization was induced using IL-4 and IL-13 cocktails. As a result, CeNL-modified and YeNL-modified macrophages displayed distinct luminescence profiles in response to M1 and M2 stimuli. Data was visualized using spectral phasor plots. This study represents a successful example of the development of a reporter system for macrophage phenotype determination and highlights the growing importance of technologies aimed at monitoring macrophage functional changes in response to environmental factors.

The study of macrophage phenotype switching itself is beyond the scope of our review, however, one study deserves attention because it involves the use of the M2 promoter. One example is the development of antitumor macrophages named MacTrigger [108,109]. In this study, murine RAW264.7 macrophages were genetically modified with plasmids encoding the *TNF-α* gene under the control of the arginase-1 (*Arg1*) promoter. As a result, the engineered macrophages migrate to the tumor site, where signals derived from the tumor microenvironment induce the expression of *Arg1* that should induce protumoral M2 polarization [108]. However, activation of the *Arg1* promoter at the same time induces expression of the *TNF-α* gene because it is located under *Arg1* promoter control. Thus, locally produced *TNF-α* resists the tumor-induced M2 polarization that would normally happen in response to microenvironmental signals and maintains a necessary level of intratumoral inflammation. This stable inflammation promotes antitumor activity and suppresses tumor progression [109]. Modulation of macrophage phenotype is a promising target for therapy of various diseases including diseases with chronic inflammation, neurodegenerative diseases, and cancer. Genetically modified macrophages have also emerged as promising therapeutic agents for targeted immunotherapy. Genetically modified macrophages have also emerged as promising therapeutic agents for targeted immunotherapy.

Among alternative methods for macrophage phenotype assessment, electrical impedance monitoring has also attracted attention. The use of cellular electrical impedance measurements for real-time monitoring of cell behavior was first introduced by Giaever and Keese in 1993 [110]. This technique was originally developed to monitor cell adhesion in real time and is based on changes in ion current flow through the cell membrane following attachment to electrode-containing surfaces.

Impedance monitoring has subsequently been applied to study differentiation of non-polarized M0 macrophages from monocytes following stimulation with PMA, with phase-contrast microscopy control as an independent validation method. Significant differences in electrical impedance values were also observed between M1 and M2 macrophages, based on differences in cell morphology and physiology. Total impedance values of M1 macrophages were more than two times higher than those observed for M2-polarized macrophages [111]. In this study, macrophage polarization was confirmed by measuring expression of marker genes after the influence of the polarization stimuli. However, this impedance-based approach was validated only in THP-1 cells and has not yet been evaluated using primary human macrophages.

In 2015, a methodology for monitoring polarization of bone marrow-derived macrophages based on their metabolic characteristics was described [112]. This approach employed multiple sensors to simultaneously monitor extracellular acidification rates (ECAR) and oxygen consumption rates (OCR), thereby providing an integrated approach of macrophage metabolic reprogramming. M1 macrophages exhibited reduced mitochondrial activity and increased glycolytic metabolism, whereas M2 macrophages demonstrated enhanced oxidative phosphorylation and increased spare respiratory capacity [112]. These findings converge with previous data showing elevated mitochondrial activity correlating with anti-inflammatory functions of M2 macrophages and therefore proves the validity of this analytical approach [113] (GalvÃ¡n-PeÃ±a & Oâ€TMNeill Galván-Peña & O’Neill, 2014). Nevertheless, despite its high sensitivity and accuracy, metabolic profiling requires noticeable financial investment and experimental time, limiting its applicability for high-throughput screening applications.

In 2007, researchers from the Institute of Basic Medical Sciences in Oslo described a reporter system where the luciferase gene was cloned under the control of the transcription factor *NF-kB* promoter [114]. THP-1 cells were transfected by electroporation and used to study the effects of adiponectin on *NF-kB* activity by measuring luminescence intensity. The study demonstrated that increasing concentrations of adiponectin enhanced *NF-kB* activity and as a result increased reporter luminescence, suggesting that adiponectin has pro-inflammatory effects in monocytic cells. This work provided a proof-of-concept for the use of reporter genes to monitor promoter activity associated with macrophage polarization.

Another recently described system for monitoring macrophage polarization is based on the use of fluorescent gold nanoparticles (AuNPs) [115]. In this system, macrophage phenotypes are identified through interactions between fluorescent probes linked to gold nanoparticles with mRNAs associated with M1 or M2 polarization. Fluorescent probes are attached to nanoparticles through complementary interaction with oligonucleotide sequences designed to recognize target mRNAs. Initially, fluorescence probes are suppressed by the gold nanoparticle. After binding to target mRNA molecules, the fluorophore becomes spatially separated from the nanoparticle surface, resulting in restoration of fluorescence emission. Fluorescence signals were analyzed using confocal microscopy. This approach has only been validated in murine RAW264.7 macrophages, whereas studies using human macrophages have not yet been published, that limits its immediate applicability for patient-derived cells.

Overall, the review describes multiple test systems for discovering macrophage polarization toward pro-inflammatory or anti-inflammatory phenotypes (Table 2). The majority of reporter systems are based on bioluminescence. Nevertheless, use of exogenous substrates may potentially have cytotoxic effects. Furthermore, the majority of published macrophage reporter systems have been developed exclusively using murine RAW264.7 macrophages and murine promoters. The small number of systems developed for human macrophages have generally relied on changes in cellular physiology rather than reporter gene expression and therefore do not provide direct reporter signals. Moreover, these systems have primarily been evaluated in THP-1 cells, whereas studies using primary human macrophages are published only for the system with metabolic profiling.

## 8. Limitations and Challenges

### 8.1. Limitations of Current Methodologies

Despite the progress in the field of development of test systems for macrophage polarisation determination there are still plenty of difficulties in macrophage phenotype monitoring. Such difficulties may affect reproducibility of the experiment results and extrapolation of in vitro results to practical in vivo research.

Current assays differ from each other by macrophage origin, detection methods, polarization protocols and marker selection and it may be difficult to compare these results. Also it is extremely challenging to consider tissue and tumor microenvironment for improving the accuracy of research.

It should be noted that, although numerous macrophage subtypes and intermediate activation states have been described in recent years, the experimental techniques currently used to investigate macrophage polarization have not yet fully validated all existing phenotypes. Most existing test systems are based on the detection of a limited number of surface markers, soluble factors and transcriptional profile and therefore primarily differentiate between the classical M1 and M2 activation states. However, accumulating data from transcriptomic, proteomic, and single-cell sequencing studies indicates that macrophages exist as a continuum of functionally different phenotypes rather than discrete populations [29]. Many newly identified macrophage subsets do not yet have universally accepted marker combinations or functional validation, making their routine identification difficult using conventional methods such as flow cytometry, immunohistochemistry, or reporter-based assays. Therefore, while existing test systems provide valuable tools for investigating macrophage biology, they currently capture only a subset of the full phenotypic diversity of macrophages observed in vivo. The next logical advance in this field will likely be the creation of multicolor, multiparametric M1/M2 assay systems capable of defining the direction of macrophage polarization.

### 8.2. Variability in Polarization Protocols

One of the most important barriers in accurate macrophage phenotype research is the lack of standardized polarization protocols. Protocols are different for macrophages from different sources, including differentiation factors, concentrations of polarization factors and exposure times. It was revealed that monocyte-derived macrophages, bone marrow-derived macrophages, tissue-resident macrophages, and immortalized cell lines show different transcriptional and functional responses to identical stimulus and there is a need for using standardised protocols at least for primary cells [29].

Different research teams use slightly different protocols of macrophage polarization and concentrations of cytokines and incubation duration influence experimental results. For example, it was shown that resident inflammatory monocyte-derived macrophages react to polarization stimuli stronger and faster than other types of macrophages that confirms the necessity of universalization and adjustment of protocols, taking into account the origin of macrophages [116].

It is also important to take into account the imperfection and simplification of the M1/M2 macrophage polarization paradigm. Of course, such a model is convenient for research, but it is worth considering the phenotypic overlap depending on their tissue location and type of signaling [73].

### 8.3. Difficulty in Translating Findings to In Vivo Settings

Another big challenge for monitoring and determination of macrophage polarization is the difficulty in extrapolating in vitro data. Macrophage polarization in tissues and tumors is influenced by numerous microenvironment factors that makes development of a real-time test system for primary cells an important problem due to simplification of conventional two-dimensional culture systems and high plasticity of macrophages [58].

One of the most illustrative examples of complex influence on macrophage phenotype is TAMs. TAMs are being influenced by a number of factors including influence of tumor-derived cytokines, hypoxia, interaction with stromal immune cells etc. and the influence of all these factors is often hard to determine exactly and even more modulate in vitro.

One of the most promising approaches for studying macrophage polarization dynamics involves the use of fluorescent reporter systems. In such systems, macrophages are genetically modified with constructs where the promoter region of a polarization-associated gene activates the expression of a fluorescent reporter protein. Changes in macrophage polarization therefore lead to changes of the reporter gene expression and corresponding changes in fluorescence intensity. This method is based on visualization of polarization pathway activation in real time and allows quantitative analysis.

Compared with bioluminescence imaging assays based on luciferase expression and oxidation of the luciferin substrate, fluorescence-based systems provide stronger signals and make possible visualization of cells located deep within tissues as well as imaging with higher resolution suitable for quantitative analysis [117]. Furthermore, bioluminescence requires constant adding of an exogenous substrate, which complicates experimental design during long-term monitoring of phenotypic changes, especially in in vivo studies. However, fluorescent reporter systems also possess several limitations. In particular, they require external excitation light sources, which may induce phototoxicity through the generation of reactive oxygen species and subsequent cellular damage [118].

### 8.4. Challenges in Defining a Clear Dichotomy Between M1 and M2 Phenotypes

One of the most controversial topics discussed in the context of macrophage biology is characterisation of their functional state based on the M1/M2 polarization paradigm. Undoubtedly M1/M2 classification turned out as extremely useful for experimental studies and general understanding of macrophage biology. Nevertheless, nowadays a lot of data proving inconsistency of the model and that the change in the phenotype is not dichotomous but distributed across the spectrum appears.

Transcriptomic studies have demonstrated that macrophage polarization is accompanied by substantial changes in gene expression that determine macrophage functional state [119]. Basic transcriptomic studies have shown that macrophages stimulated by different polarization factors demonstrate a wide range of activation states that cannot be fully explained by the classical M1/M2 polarization model. In contrast, macrophage polarization represents a range of phenotypes characterized by both shared and stimulus-specific patterns of gene expression. Also, this study has identified transcriptional regulators involved in both general macrophage activation and the development of specific macrophage phenotypes and their connection to each other.

TAMs are one of the examples of “mixed phenotype” demonstration. Tumor type and disease stage influence expression of pro- and anti-inflammatory markers in TAMs that was proved by gene expression, cellular morphology, and cytokines profile analysis [120].

**Figure 1:**
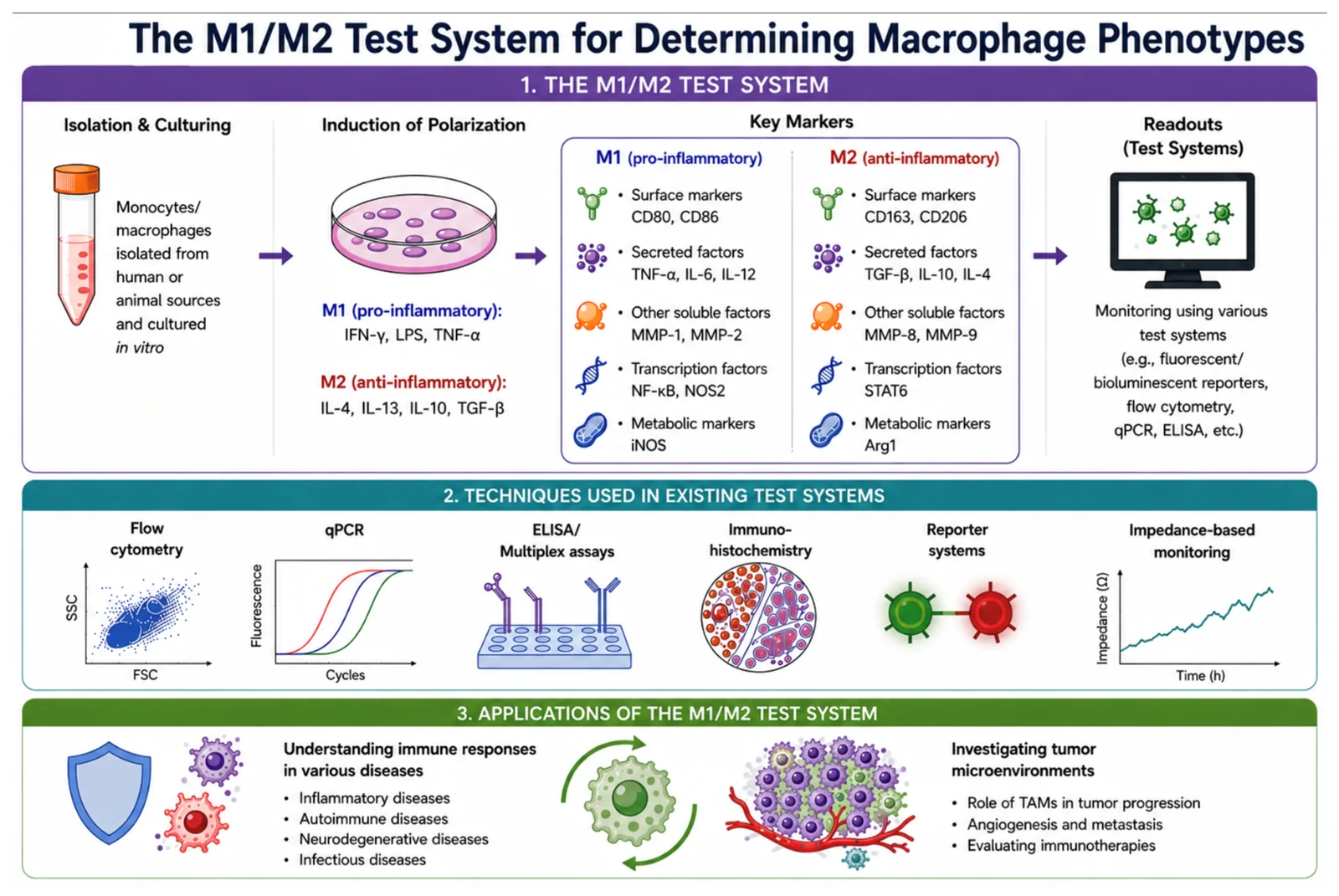
Overview of M1/M2 macrophage phenotype determination: markers, test systems, and applications. Arrows indicate the stages of the M1/M2 test system pipeline, from macrophage isolation and culturing through polarization and phenotype characterization to the final readout. The figure was generated using ChatGPT (GPT-5.6 Sol, OpenAI, 2026) with the integrated image-generation tool, based on detailed prompts provided by the authors regarding the structure, scientific content, labels, and visual organization of the figure. The generated image was subsequently reviewed and revised by the authors to ensure the accuracy of all scientific information presented.

All things considered, using a small number of markers for macrophage phenotype determination may lead to missing out important functional signatures and inaccurate interpretation of data (Figure 1). Modern research is based not only on common polarization markers analysis but also uses transcriptomic, proteomic and metabolomic data to take into account all the functional features of the cells.

The development of omics technologies has significantly changed the understanding of macrophage phenotypes. While the classical M1/M2 model remains widely used, transcriptomic studies demonstrate that macrophage activation represents a spectrum of functional states, not only two polar phenotypes. In particular, analysis of human macrophages in response to different stimuli showed distinct and partially overlapping transcriptional programs, demonstrating that macrophage phenotype depends significantly on the signals of the microenvironment [119,121].

The appearance of single-cell methods, specifically single-cell RNA sequencing, has improved and expanded macrophage phenotype concept and made it possible to investigate macrophage functional state at the level of individual cells. Transcriptomic analysis provides an average gene-expression profile of a cell population, while single-cell RNA sequencing can identify transcriptionally different macrophage populations in the same tissue. These technologies are especially useful for TAMs that are exposed to heterogeneous combinations of cytokines, metabolites, hypoxia, tumor-derived molecules, and signals from other immune cells [121]. Research of TAM showed that tumor-associated macrophages often exhibit transcriptional programs that combine characteristics traditionally related to both M1 and M2 macrophages and cannot be accurately classified using the classical binary M1/M2 paradigm [122].

In addition to single-cell RNA sequencing, spatial transcriptomic, proteomic and metabolomic methods expanded understanding of changes in functional state of macrophages. Spatial transcriptomics gives an opportunity to connect transcriptional states data with specific tumor regions, which is especially important for TAMs [123]. Proteomic technologies can identify changes in receptors, signaling proteins, enzymes, and mediators, whereas metabolomic technologies describe functional changes in pathways such as glycolysis and oxidative phosphorylation [123].

It is important to note that multi-omics methods reveal the complexity of changes of macrophage phenotype but do not eliminate the practical value of the M1/M2 model. The M1/M2 classification is useful for standardized in vitro experiments, especially when macrophages are subjected to different polarization stimuli and when the goal is to compare predominantly pro-inflammatory and anti-inflammatory responses. According to this, M1/M2 test systems provide useful information about the direction and dynamics of macrophage polarization.

## 9. Future Directions

A strict M1/M2 paradigm is undoubtedly an oversimplification of the complex, continuously dynamic, and balanced macrophage state. Nevertheless, this terminology has become firmly established and remains widely used in articles published in high-impact journals [124]. A range of high-technology approaches is currently available for defining macrophage phenotypes, although some have not yet been widely adopted. These include single-cell omics technologies, particularly single-cell RNA sequencing, as well as single-cell mass cytometry. Using the latter, the seminal work by Slavov and colleagues demonstrated that macrophages exhibit spontaneous polarization even in the absence of external stimuli [30]. A potential use case for the reporter system discussed in this review, together with single-cell RNA sequencing, is profiling the same cells by promoter activity and their transcriptomic trajectories. Label-free biophysical measurements also represent a promising direction and may be combined with FLIM-compatible sensor-based macrophage test systems, which have not yet been described in the literature.

The integration of these technologies will enable the classification of macrophages beyond bulk M1/M2 averages, moving toward transcriptionally defined trajectories and gene regulatory networks. Combining single-cell RNA sequencing with spatial transcriptomics, advanced microscopy, and test systems will allow simultaneous assessment of macrophage state, localization, and dynamics within intact tissues in animal models. This approach will also make it possible to evaluate the relationship between polarization signatures and cell-cell interactions, migration, and phagocytosis, rather than relying on discrete analyses of isolated or fixed cells.

The application of combined techniques, together with the increasing resolution of single-cell analyses in human tissues, will allow M1/M2 test systems to evolve into clinically oriented assays that integrate surface markers, secreted factors, and transcriptional profiles, enabling monitoring of how macrophage programs shift during therapy. In parallel, ex vivo test systems based on patient-derived monocytes or tissue-resident macrophages could be used to assess responses to drugs, effectively functioning as companion diagnostics for therapy selection.

## 10. Conclusions

Macrophages play a central role in intersection of inflammation, tissue repair, and tumor progression, and their remarkable plasticity underlies both protective and pathological responses in a wide range of diseases. The conventional M1/M2 paradigm remains useful, but it fails to capture the continuous spectrum of activation states revealed by modern transcriptomic and proteomic analyses, particularly in complex settings such as tumor-associated or chronically inflamed tissues.

Existing M1/M2 test systems, built around analysis of surface and secreted markers, and endpoint functional assays, have greatly facilitated mechanistic studies and drug testing but are limited by poor standardization, model-dependent responses, and a predominant reliance on murine or immortalized cell lines. Emerging technologies—including bioluminescent and fluorescent reporter systems, electrical impedance and metabolic profiling, and other label-free biophysical approaches—demonstrate that real-time monitoring of macrophage phenotype dynamics is feasible and can reveal transient and hybrid states that are invisible to static assays.

Future progress will depend on integrating these test systems with single-cell and spatial omics, as well as with ex vivo models based on patient-derived monocytes and tissue-resident macrophages, to generate clinically relevant macrophage phenotyping platforms. Such integrated M1/M2 test systems have the potential to serve not only as research tools, but also as companion diagnostics for stratifying patients, predicting therapeutic responses, and guiding macrophage-targeted interventions in cancer, autoimmune and infectious diseases.

## Figures and Tables

**Table 1:** Overview of markers associated with M1 and M2 macrophage polarization. The most commonly used markers are highlighted in bold.

	Pro-Inflammatory M1-Macrophages	Anti-Inflammatory M2-Macrophages
Surface markers	**CD80**, **CD86**, CD40, CD64, CCR7, TLR2, TLR4, MHC-II, HLA-DR	**CD163**, **CD206**, CD204, CD209
Secreted factors	***TNF-α***, **IL-6**, **IL-12**, IL-1α, **IL-1β**, IL-18, IL-23, IFN-γ, CXCL1, CXCL3, CXCL5, CXCL8, CXCL9, CXCL10, CXCL11, CXCL16; CCL2, CCL3, CCL5, CCL8, CCL15, CCL19, CX3CL1	**TGF-β**, **IL-10**, IL-4, IL-13, IL-33, CCL1, CCL2, CCL13, 1, CCL17, **CCL18**, CCL22, CCL24, CCL26
Other soluble factors	MMP-1, MMP-3, MMP-7, MMP-10, MMP-25, LTB4, TXB2	MMP-8, MMP-9, MMP-11, MMP-12, MaR1, RvD5, RvE2, PD1, LXA4, FIZZ1, Ym1/2

**Table 2:** Overview of existing methods and test systems for identification of macrophage phenotype.

Test System	Operating Principal	Polarization-Associated Markers	Detection Principal	Cell Model
*Arg1*-luciferase reporter macrophages for phenotype identification in vitro and in vivo in tumors	Activation of the M2-associated *Arg1* promoter by IL-4 induces expression of luciferase	*Arg1* (M2-associated marker)	Detection of luminescence to M2 phenotype using a luminometer	Murine RAW264.7 macrophages
BRET macrophage reporter system	Synchronous monitoring of M1 and M2 promoter activity using spectral separation	*NOS2* (M1), *STAT6* (M2)	Bioluminescence resonance energy transfer (BRET) using CeNL and YeNL nanoluciferases and phasor analysis	Murine RAW264.7 macrophages cultured in 3D collagen matrices
Electrical impedance monitoring	Changes in cell morphology and adhesion modify electrical impedance	No direct molecular markers	Electrical impedance measurement using electrode-coated culture plates	Human THP-1 macrophages
Metabolic profiling (ECAR/OCR analysis)	Macrophage polarization is associated with metabolic reprogramming	Glycolytic activity and mitochondrial respiration	Measurement of extracellular acidification rate (ECAR) and oxygen consumption rate (OCR)	Murine bone marrow-derived macrophages
*NF-kB*-luciferase THP-1 reporter system	Activation of inflammatory signaling pathways induces NF-κB activity	*NF-kB* (M1-associated transcription factor)	Luciferase expression under control of *NF-kB* transcription factor	Human THP-1 macrophages
Gold nanoparticle (AuNP)-based reporter system	Hybridization of target mRNA releases silenced fluorophore from nanoparticle surface	M1- and M2-associated mRNAs	Fluorescence recovery following target mRNA binding	Murine RAW264.7 macrophages
Routine endpoint phenotyping approaches	Quantification of polarization markers at certain time points	Surface markers (CD80, CD68, CD163, CD206), cytokines, gene expression markers	Flow cytometry, ELISA, qPCR, Western blotting	Cell lines and primary macrophages

## Data Availability

No new data were created or analyzed in this study. Data sharing is not applicable to this article.

## Abbreviations

The following abbreviations are used in this manuscript:

*Arg1*	Arginase 1
AuNP	Gold nanoparticle
BM	Bone marrow
BMDM	Bone marrow–derived macrophage
BRET	Bioluminescence resonance energy transfer
CCR	C-C motif chemokine receptor
CCL	C-C motif chemokine ligand
CeNL	Cerulean-based nanoluciferase
CXCL	C-X-C motif chemokine ligand
DC	Dendritic cell
ECAR	Extracellular acidification rate
ELISA	Enzyme-linked immunosorbent assay
FIZZ1	Found in inflammatory zone 1
GM-CSF	Granulocyte–macrophage colony-stimulating factor
HIF-1α	Hypoxia-inducible factor 1 alpha
IHC	Immunohistochemistry
iNOS/*NOS2*	Inducible nitric oxide synthase
LPS	Lipopolysaccharide
M-CSF	Macrophage colony-stimulating factor
MHC-II	Major histocompatibility complex class II
NF-κB	Nuclear factor kappa-light-chain-enhancer of activated B cells
OCR	Oxygen consumption rate
PBMC	Peripheral blood mononuclear cell
PMA	Phorbol 12-myristate 13-acetate
PAMP	Pathogen-associated molecular pattern
qPCR	Quantitative polymerase chain reaction
RAW264.7	Murine macrophage cell line RAW 264.7
*STAT6*	Signal transducer and activator of transcription 6
TAM	Tumor-associated macrophage
TGF-β	Transforming growth factor beta
THP-1	Human monocytic cell line THP-1
TLR	Toll-like receptor
